# Dissecting Interlayer Hole and Electron Transfer in Transition Metal Dichalcogenide Heterostructures via Two-Dimensional Electronic Spectroscopy

**DOI:** 10.1021/acs.nanolett.1c01098

**Published:** 2021-05-26

**Authors:** Veronica R. Policht, Mattia Russo, Fang Liu, Chiara Trovatello, Margherita Maiuri, Yusong Bai, Xiaoyang Zhu, Stefano Dal Conte, Giulio Cerullo

**Affiliations:** † IFN-CNR, Dipartimento di Fisica, Politecnico di Milano, 20133 Milano, Italy; ‡ Department of Chemistry, Stanford University, Stanford, California 94305, United States; § Department of Chemistry, Columbia University, New York, New York 10027, United States

**Keywords:** transition metal dichalcogenides, van der Waals heterostructure, interlayer charge transfer, ultrafast spectroscopy

## Abstract

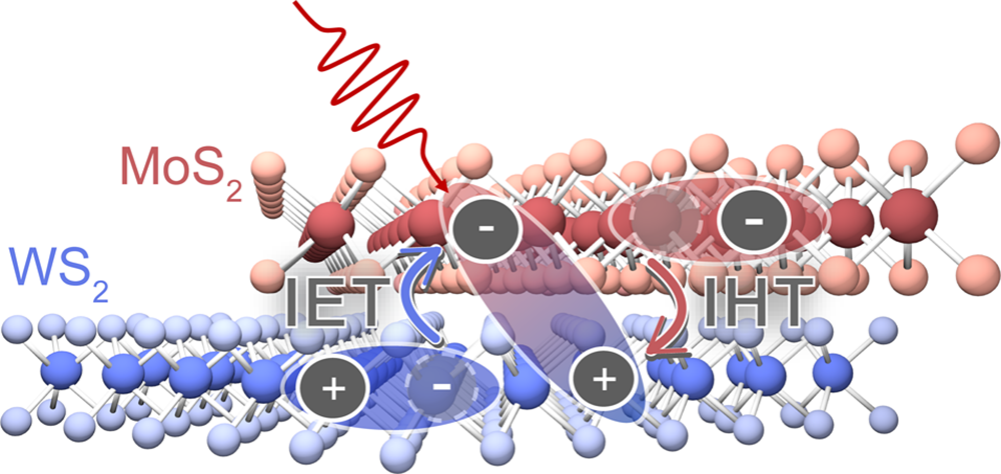

Monolayer transition metal dichalcogenides (ML-TMDs) are two-dimensional semiconductors that stack to form heterostructures (HSs) with tailored electronic and optical properties. TMD/TMD-HSs like WS_2_/MoS_2_ have type II band alignment and form long-lived (nanosecond) interlayer excitons following sub-100 fs interlayer charge transfer (ICT) from the photoexcited intralayer exciton. While many studies have demonstrated the ultrafast nature of ICT processes, we still lack a clear physical understanding of ICT due to the trade-off between temporal and frequency resolution in conventional transient absorption spectroscopy. Here, we perform two-dimensional electronic spectroscopy (2DES), a method with both high frequency and temporal resolution, on a large-area WS_2_/MoS_2_ HS where we unambiguously time resolve both interlayer hole and electron transfer with 34 ± 14 and 69 ± 9 fs time constants, respectively. We simultaneously resolve additional optoelectronic processes including band gap renormalization and intralayer exciton coupling. This study demonstrates the advantages of 2DES in comprehensively resolving ultrafast processes in TMD-HS, including ICT.

Semiconducting monolayer transition metal dichalcogenides (ML-TMDs) are a class of two-dimensional crystalline materials with enticing electronic and optical properties,^1,2^ including a direct band gap in the visible range, strong light–matter coupling, enhanced excitonic correlations, and spin/valley locking.^3−5^ A growing area of TMD research involves vertical stacks of ML-TMDs, referred to as TMD heterostructures (HSs).^6,7^ Weak interlayer van der Waals forces between ML-TMDs bypass the lattice parameter matching constraints of conventional semiconductor HSs and preserve the electronic structure of each constituent, enabling the creation of new materials with tailored electronic and optical properties which differ from those of the isolated MLs.

Several TMD-HSs present a type II (staggered) band alignment, in which the valence band (VB) maximum and the conduction band (CB) minimum at the K/K′ points reside in different layers. This alignment favors charge separation and the formation of interlayer (IL) excitons via interlayer charge transfer (ICT). IL excitons are of particular interest as they feature recombination time scales (i.e., 1–100 ns)^8−10^ orders of magnitude longer than those of intralayer excitons in isolated ML-TMDs (i.e., ∼100 ps).^11^ The strong enhancement of the IL exciton lifetime, due to a reduced spatial overlap of the electron and hole wave functions, is responsible for the observation of several phenomena including the diffusion of IL excitons over micrometer length scales^12,13^ and extremely long valley polarization retention,^14,15^ among others. Enhanced IL exciton lifetimes make TMD-HSs with type II band alignment excellent candidates for optoelectronic and light-harvesting applications.^16^

A variety of spectroscopic techniques, including photoluminescence and ultrafast transient absorption (TA) spectroscopy, have revealed ICT in TMD-HSs^17,18^ to be extremely rapid^19−26^ and very efficient.^23^ ICT has been previously reported to occur on a sub-100 fs time scale,^19−26^ though there remains uncertainty in the rates of ICT and a lack of information about differences in electron versus hole transfer times within the same system. Several studies on IL electron transfer have used above-resonance excitation, which introduces additional many-body effects into the system, and it is unclear how to disentangle these effects from ICT processes. Experimental studies show that the time scales and efficiencies of ICT are independent of the IL twist angle.^21,24,25,27^ Several theories as to how ICT proceeds without hindrance by interlayer momentum mismatch include mediation via local structural inhomogeneity,^25^ excess electronic energy,^21,23^ phonon-mediated intermediate scattering through hybridized valleys,^26,28−32^ and long-lived quantum coherences at the interface.^33^

Obtaining a comprehensive picture of ICT processes in TMD-HSs is of critical importance both for understanding the fundamental physics at play and for developing optoelectronic applications. One key experimental limitation in ultrafast TA studies of ICT thus far has been the trade-off between temporal and spectral resolution. Ultrafast TA spectroscopy is limited in its ability to distinguish between transitions in spectrally congested systems, where electronic transitions are close in energy. In fact, spectral selection of one transition requires narrowband pump pulses which, due to the Fourier transform (FT) limitation, reduce the temporal resolution of the experiment.

Two-dimensional electronic spectroscopy (2DES) is a multidimensional spectroscopy technique that measures the third-order material polarization using a sequence of three pulses: two excitation pulses separated by the so-called coherence time, *t*_1_, and a detection pulse that is delayed by the waiting time, *t*_2_, with respect to the second excitation pulse (Figure 1a). FT over the coherence time *t*_1_ allows for resolution over the excitation frequency axis, while resolution over the detection frequency axis is typically obtained by dispersing the detection pulse in a spectrometer.^34−36^ 2DES can obtain high excitation frequency resolution while using broadband pulses and thus simultaneously maximize spectral and temporal resolution. 2DES is very well-suited for studying ultrafast dynamics in systems with a high degree of spectral congestion^37^ and has been exploited to measure inhomogeneous/homogeneous line widths,^38−41^ excitonic coupling,^42−44^ exciton valley coherence,^39^ and biexcitons^38^ in ML-TMDs.

**Figure 1:**
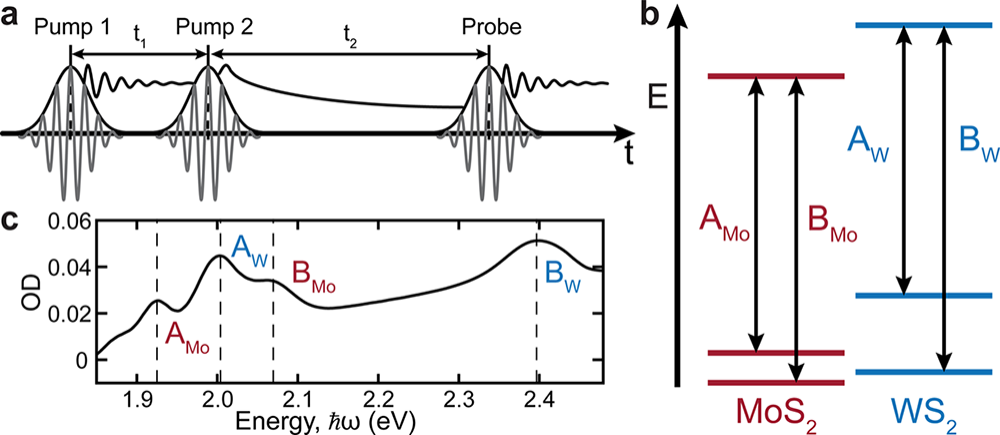
(a) 2DES pulse scheme. Two pump pulses are separated by the coherence time, *t*_1_, and are followed by a probe pulse delayed by the waiting time, *t*_2_. Scanning and Fourier transforming *t*_1_ yields the excitation frequency axis (ω_1_). (b) Electronic energy levels of the A and B excitons of the WS_2_/MoS_2_ HS. (c) 80 K linear absorption spectrum of the WS_2_/MoS_2_ HS with labeled peaks for the A and B excitons.

Here, we use 2DES to simultaneously measure both interlayer electron and hole transfer dynamics in a large-area WS_2_/MoS_2_ HS prepared using a novel mechanical exfoliation technique.^45^ This is achieved by using extremely short (sub-20 fs) and broadband pulses with spectra covering the A and B exciton of MoS_2_ and the A exciton of WS_2_. We observe signatures of intra- and interlayer coupling between the A/B excitons of MoS_2_ and the A exciton of WS_2_ immediately after photoexcitation. We unambiguously resolve interlayer hole transfer (IHT) to WS_2_ following selective excitation of the A exciton of MoS_2_ with a 34 ± 14 fs time constant and furthermore resolve interlayer electron transfer (IET) to MoS_2_ following excitation on resonance with the A exciton of WS_2_ with a 69 ± 9 fs time constant. We corroborate our findings by comparing these results with 2DES measurements of the individual layered components of the HS.

Figure 1b reports a sketch of the band alignment at the K point for the WS_2_/MoS_2_ HS as calculated in ref (46). For clarity, we identify the A and B excitons of each layer of the HS with a subscript corresponding to the layer’s transition metal (A_X_ and B_X_ where X = Mo or W). Single crystal millimeter-scale ML-TMDs are prepared via gold-tape exfoliation of bulk crystals.^45^ The large-area WS_2_/MoS_2_ HS is obtained by vertically stacking the MLs and transferring them onto a transparent 200-μm-thick SiO_2_ substrate;^45^ an interlayer twist angle of 29.5 ± 0.9° was determined by polarization-resolved second harmonic generation measurements (Figure S3). The absorption spectrum of the HS at 80 K is shown in Figure 1c. The 2DES experiments are performed at 80 K using a partially collinear pump–probe geometry and measuring the real absorptive third-order nonlinear signal. The home-built 2DES instrument generates the pump–pulse pair with birefringent wedges, which scan the coherence time, *t*_1_, with high phase stability.^47^

2DES maps plot the correlation between excitation energy (ℏω_1_) and detection energy (ℏω_3_) for a given waiting time, *t*_2_. We begin by examining the 2DES maps of the real absorptive signal of the ML-MoS_2_ (Figure 2a). The 2DES map at *t*_2_ = 0 displays two positive diagonal (ℏω_1_ = ℏω_3_) peaks corresponding to photobleaching (PB) of A_Mo_ and B_Mo_ excitons. At early times, the diagonal peaks are inhomogeneously broadened along the diagonal due to the spatial heterogeneity of the sample, while the homogeneous line width lies along the antidiagonal direction. Spectral signatures of the off-diagonal or cross-peaks (ℏω_1_ ≠ ℏω_3_) between A_Mo_ and B_Mo_ at *t*_2_ = 0 fs indicate strong intralayer excitonic coupling, whereas the *t*_2_-dependent behavior reports on intralayer exciton dynamics and coupling strengths. Strong coupling between the A and B excitons in ML-MoS_2_^42,44,48^ and other ML-TMDs^49^ has been previously observed and ascribed to several mechanisms: light-induced band gap renormalization (BGR),^48^ Dexter-like intervalley exciton coupling,^50^ and intervalley exchange interaction,^42^ among others.^49,51,52^ At later times, all four peaks exhibit horizontal elongation along the excitation axis, consistent with previous observations, using ultrafast TA, of a delayed excitonic PB following above-resonance excitation.^53^

**Figure 2:**
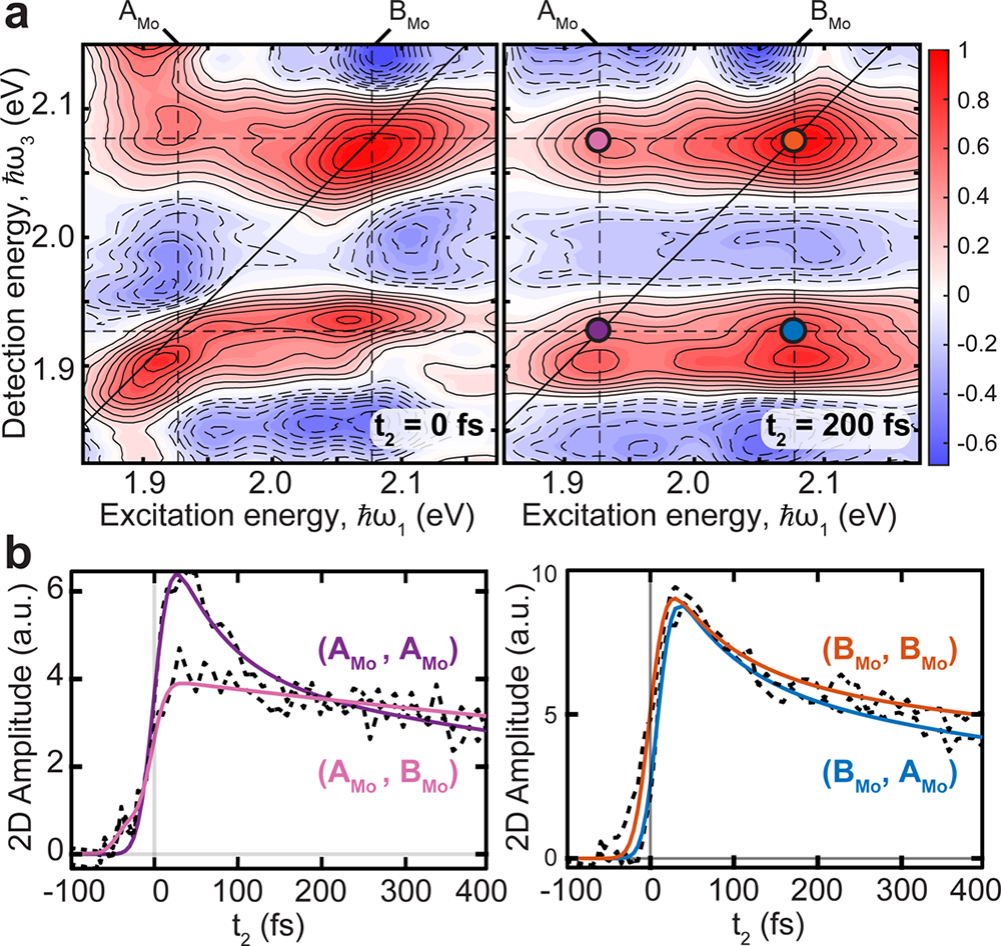
(a) 2DES maps of the real absorptive signal of ML-MoS_2_ at 80 K at *t*_2_ = 0 fs (left) and 200 fs (right). Positive (negative) sign signals are red (blue) with filled (dashed) contours drawn in 10% increments of the maximum. The incident pump fluence is 10 μJ/cm^2^. Dashed lines indicate the energies of A_Mo_ (ω = 1.92 eV) and B_Mo_ (ω = 2.07 eV) excitons. (b) Values of *t*_2_ dynamics of MoS_2_ ML following excitation of the A_Mo_ (left) and B_Mo_ excitons (right). The *t*_2_ traces are shown with fits color coded for dots in (a).

The early temporal dynamics of the ML-MoS_2_ excitonic signatures are plotted as a function of waiting time, *t*_2_, in Figure 2b. With the exception of the above-diagonal cross-peak at (ℏω_1_,ℏω_3_) = (A_Mo_,B_Mo_) (pink circle), the signatures display an instantaneous rise and a double exponential decay within the 1 ps temporal window of the measurement. The ultrafast decay is consistent with previous observations and has been attributed to carrier thermalization and cooling.^54^ The above-diagonal cross-peak is characterized by a slightly delayed PB formation followed by a single exponential decay. Similar dynamics of the (A_Mo_,B_Mo_) peak were recently reported in a 2DES study of chemical vapor deposition grown ML-MoS_2_ using a cocircularly polarized configuration and attributed to an excitonic exchange interaction.^42^ Negative peaks in the 2D maps (blue, Figure 2a) are due to photoinduced absorption and are located at detection energies below the positive PB peaks. These features are attributed to many-body effects which are referred to cumulatively as BGR.^44,48^

The 2DES maps of the WS_2_/MoS_2_ HS (Figure 3a) show many distinct features compared to the ML-MoS_2_. At *t*_2_ = 0 fs, we see prominent diagonal peaks of the A_Mo_ and B_Mo_ PB, as in the ML-MoS_2_ sample, with an additional diagonal peak from the A_W_ exciton. The A_W_ peak is red-shifted in comparison to the A_W_ of the ML-WS_2_ (Figure S4) and is well separated spectrally from the A_Mo_ and B_Mo_ excitons. We attribute the spectral shift of A_W_ to differences in the dielectric environment of the WS_2_ layer in the HS.^55,56^ At *t*_2_ = 0 fs, the WS_2_/MoS_2_ HS 2DES maps show significant broadening along the diagonal, due to overlap of the three inhomogeneously broadened excitons in close spectral proximity to one another. Similar to ML-MoS_2_, Figure 3a shows signatures of strong intralayer coupling between the A_Mo_ and B_Mo_ excitons, resulting in cross-peaks present at *t*_2_ = 0 fs. We are unable to resolve intralayer cross-peaks of the WS_2_ layer as the B_W_ exciton falls outside our spectral detection window.

**Figure 3:**
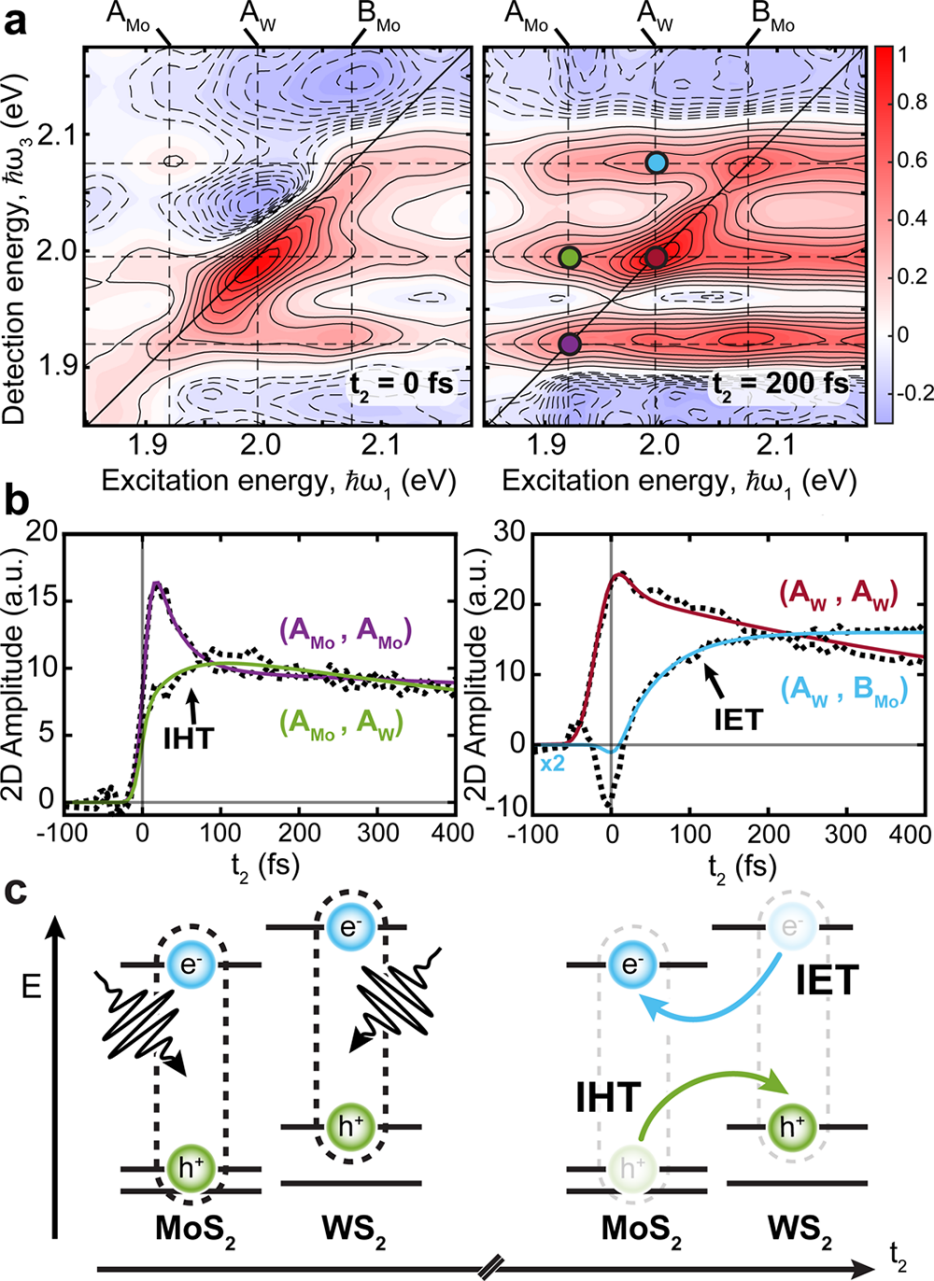
(a) 2DES maps of the real absorptive signal of WS_2_/MoS_2_ HS at 80 K for *t*_2_ = 0 fs (left) and 200 fs (right). Dashed lines indicate the energies of A_Mo_ (ℏω = 1.92 eV), A_W_ (ℏω = 1.99 eV), and B_Mo_ (ℏω = 2.07 eV) excitons. The pump incident fluence is 13 μJ/cm^2^. The photoinduced carrier density is estimated to be ∼5.5 × 10^12^ cm^–2^ by considering the absorption coefficients of the individual layers. (b) Values of *t*_2_ dynamics of ICT signatures. Signatures of IHT following excitation of the A_Mo_ exciton (left) show a delayed rising component at the detection energy of A_W_. Signatures of IET following excitation of the A_W_ exciton (right) show a delayed rising component at the detection energy of B_Mo_. The *t*_2_ traces are shown with fits color coded for dots in (a). The (A_W_,B_Mo_) *t*_2_ trace is multiplied by a factor of 2 for better comparison. (c) Cartoon illustrating the process of interlayer hole and electron transfer upon optical excitation of the MoS_2_ and WS_2_ layers, respectively.

We can identify several spectral signatures unique to the HS which are the result of interlayer coupling and ICT processes. Several below-diagonal signatures of interlayer coupling are visible at *t*_2_ = 0 fs at (ℏω_1_,ℏω_3_) = (B_Mo_,A_W_) and (A_W_,A_Mo_). These signatures show an instantaneous rise similar to the diagonal peaks (Figure S7). At later times, additional interlayer cross-peaks grow in above the diagonal. The 2D map at *t*_2_ = 200 fs shows a grid of positive PB signals between every possible combination of the three excitonic states along with weak negative BGR signals red-shifted along ℏω_3_ from each excitonic resonance. The above-diagonal cross-peaks (green and cyan circles, Figure 3a) display a delayed PB signal. We assign these interlayer cross-peaks to ICT processes: IHT and IET for the peaks located at (ℏω_1_,ℏω_3_) = (A_Mo_,A_W_) and (A_W_,B_Mo_), respectively.

The delayed PB at (ℏω_1_,ℏω_3_) = (A_Mo_,A_W_) (Figure 3b) is consistent with a dissociation of A_Mo_ excitons, leading to ultrafast hole scattering from the upper VB of the MoS_2_ to the higher-energy upper VB in the WS_2_ at the K(K′) point (Figure 3c). To confirm this assignment, we compare to the 2DES data of ML-MoS_2_ and ML-WS_2_. The *t*_2_ dynamics of the ML-MoS_2_ sample taken at the same excitation/detection energy as the IHT signature shows a completely different behavior: an instantaneous buildup and a negative signal due to the BGR upon A_Mo_ resonant photoexcitation (Figure S8). The isolated ML-WS_2_ sample (Figure S4) gives rise to a negligible signal because the excitation energy is below the optical gap of the material. We can then attribute the delayed rise of the cross-peak in the HS to IHT.

The above diagonal *t*_2_ dynamics of the peak at (ℏω_1_,ℏω_3_) = (A_W_,B_Mo_) (Figure 3b) shows a slightly slower buildup of the PB signature following excitation of the A_W_ exciton, which is consistent with the dissociation of the A_W_ exciton due to the scattering of the electron from the CB of WS_2_ to the lower energy CB of MoS_2_ at the K(K′) points. In this case, however, the excitation resonant with the A_W_ exciton photoinjects electron/hole pairs into both the TMD layers, and so we expect that the PB of the cross-peak would have an additional contribution given by the combination of Pauli blocking and many-body correlation effects due to electron/hole pairs directly photoexcited above the optical gap of MoS_2_. Again, the *t*_2_ dynamics of the isolated MoS_2_ and WS_2_ layers, measured at the same excitation/emission energy as the IET signature, do not display any retarded buildup time, as reported in Figure S9. This comparison clearly shows that the *t*_2_ dynamics of the HS is not a mere sum of the dynamics of the two TMD layers, and therefore, the slow rise time of the (A_W_,B_Mo_) peak can be mostly attributed to the IET process.

To quantify the time scales of the delayed PB signatures of ICT processes, we fit the buildup *t*_2_ dynamics with the convolution between a rising exponential and a Gaussian instrument response function accounting for the finite temporal resolution of the experiment (a sum of decaying exponentials is used to fit the relaxation dynamics). More details of the fitting model and the fitting results can be found in the Supporting Information. The interlayer cross-peak at (ℏω_1_,ℏω_3_) = (A_Mo_,A_W_) is fit with a 34 ± 14 fs fast growth component and 1.2 ± 0.1 ps decay component, which is the result of intra- and interlayer recombination processes (Figure S7). A sub-100 fs time scale was tentatively claimed for hole transfer process by previous TA measurements on the same HS, but the lack of high temporal resolution hindered the precise characterization of the scattering process.^19,23,25^ The IET dynamics at (ℏω_1_,ℏω_3_) = (A_W_,B_Mo_) displays a slightly slower rise time that has been fitted to be 69 ± 9 fs (Figure S7). Recent time-resolved angle-resolved photoemission spectroscopy measurements^26^ are consistent with this time scale and show that photoexcited electrons in the K valley can spread their occupation on a broad momentum space, quickly scattering to other valleys which display strong layer hybridization. Sub-100 fs intervalley scattering of electrons in the CB and strong valley hybridization could then facilitate IET process. Slower IET time with respect to IHT was predicted, although overestimated, by theoretical calculations and attributed to the weaker donor–acceptor coupling and shorter quantum coherence.^57^ We emphasize that the accurate measurement of the ICT times in this study is only possible due to the combination of high temporal and spectral resolution of the 2DES technique. The fitting also reveals additional differences between the ML-MoS_2_ and the HS. We find that the above-diagonal intralayer cross-peak (A_Mo_,B_Mo_) in the HS shows a formation time (i.e., 90 ± 20 fs) more delayed than that in the ML-MoS_2_ (<20 fs) (see Figure S5). This effect can be explained as a result of a competition between the exchange coupling between A/B excitons of ML-MoS_2_ and the IHT process. The depletion of the A exciton population might result in a decreased interaction with the higher energy B exciton and, as a consequence, further increase of the (A_Mo_,B_Mo_) cross-peak formation time scale.

We have focused on the two above-diagonal signatures of ICT for our analysis thus far, though two additional signatures of ICT are expected below the diagonal in Figure 3a. As described above, the below-diagonal (A_W_,A_Mo_) and (B_Mo_,A_W_) peaks are characterized by pulse-width-limited buildup dynamics with overlapping signals which are determined by both intralayer and interlayer scattering processes. The absence of delayed formation in the PB peaks of the below diagonal cross-peaks is a signature that the former of these processes prevails and determines the formation dynamics. This result is supported by recent experiments showing that electron–hole pairs photoexcited above the optical gap of TMDs relax on the bottom (top) of the CB (VB) at the K point on time scale faster than 20 fs.^53^

In conclusion, we have performed broadband 2DES on a large-area WS_2_/MoS_2_ HS, covering simultaneously the A and B excitons of MoS_2_ and the A exciton of WS_2_. We unambiguously observe IHT to WS_2_ with 34 ± 14 fs time constant following excitation of the A_Mo_ exciton and IET to MoS_2_ with 69 ± 9 fs time constant following excitation of the A_W_ exciton. Our results demonstrate the ability of 2DES, due to its unique combination of high temporal and spectral resolution, to distinguish between various excitonic dynamics in a spectrally congested TMD-HSs, overcoming the limitations of conventional TA spectroscopy. Continuing to implement 2DES on different large-area TMD-HS samples will allow us to characterize the extremely rapid exciton and charge carrier dynamics and their dependence on interlayer tilt angle, among many other variables.

## Supporting Information Available

Two-dimensional electronic spectroscopy (2DES); 2DES setup; pulse characterization; large-area sample preparation and characterization of interlayer twist angle; 2DES map of isolated WS_2_; fitting procedure and time constants; supplementary data (PDF)
