# Supplementary material for: Dissecting Interlayer Hole and Electron Transfer in Transition Metal Dichalcogenide Heterostructures via Two-Dimensional Electronic Spectroscopy
Source: Nano Lett. 2021 May 26;21(11):4738–43. doi: 10.1021/acs.nanolett.1c01098 (PMC8289282; doi:10.1021/acs.nanolett.1c01098)
Supplement: Supplementary file 1 — nl1c01098_si_001.pdf [file nl1c01098_si_001.pdf]

Supporting Information

Dissecting Interlayer Hole and Electron Transfer in Transition Metal Dichalcogenide Heterostructures via Two-Dimensional Electronic Spectroscopy

Veronica R. Policht[1], Mattia Russo[1], Fang Liu[2], Chiara Trovatello[1], Margherita Maiuri[1], Yusong Bai[3], Xiaoyang Zhu[3], Stefano Dal Conte[1*], and Giulio Cerullo[1*]*

[1] IFN-CNR, Dipartimento di Fisica, Politecnico di Milano, Milano, Italy

[2] Department of Chemistry, Stanford University, Stanford, CA, USA

[3] Department of Chemistry, Columbia University, New York, NY, USA

Two-dimensional electronic spectroscopy (2DES)

Two-dimensional electronic spectroscopy (2DES) is a multidimensional Fourier transform spectroscopy method[1,2] based on its vibrational analogue, Two-dimensional Infrared Spectroscopy (2DIR), which extends to optical frequencies techniques commonly used in nuclear magnetic resonance[3]. 2DES measures the third-order nonlinear material polarization ($P^{(3)}(\omega)$) following perturbation by a sequence of three laser pulses: two pump pulses and a probe pulse (Figure 1a). This method measures the same type of signal as transient absorption (TA) spectroscopy. However, whereas TA methods employ a two-pulse sequence, pump and probe, with a single waiting time t_2 between the pulses, 2DES introduces a second pump pulse with an additional time delay t_1 between the two pumps. By scanning t_1 and performing a Fourier transform to ω_1, 2DES resolves the excitation frequency. The detection frequency (ω_3) is measured directly either by heterodyne or homodyne detection in a spectrometer. In this way, 2DES is not limited by the same pulse bandwidth/temporal resolution tradeoff as TA techniques as there is no ambiguity of the initial light absorption event when using transform-limited broadband pulses. 2DES signals can be related to TA by integration of the real absorptive signal over a portion of the ω_1 axis.

2DES generates two types of signals referred to as the rephasing and non-rephasing signals, defined by their phase matching criteria, k_R and k_{NR}:

$$k_R = -k_1 + k_2 + k_3$$

$$k_{NR} = k_1 - k_2 + k_3$$

where k_n is the wave vector of the nth pulse (pump 1 = 1, pump 2 = 2, probe = 3). The rephasing signal is analogous to a stimulated photon echo signal. Collection of both the rephasing and non-rephasing signals is necessary to reconstruct the real absorptive signal ($S_{Abs} = S_R + S_{NR}$). In a 2DES instrument with a partially collinear pump-probe beam geometry, like the one used in this

experiment[4], $k_1 = k_2$ so that both the rephasing and non-rephasing signals propagate collinearly with the probe pulse ($k_R = k_{NR} = k_3$), allowing for measurement of the absorptive signal directly along the probe path[5,6].

The inter-pump coherence time, t_1, is so named as the system evolves as an electronic coherence following the light-matter interaction of the first pump pulse. During this time, the signal oscillates with the electronic transition frequency and decays rapidly. The light-matter interaction of the second pump pulse can bring the system back into a population either of the ground or of the initially excited state or can interact with a different electronic transition, putting the system into a coherent superposition between the two excited states. The waiting time t_2 between the second pump and the probe (Figure 1a) is scanned to allow the populations and coherent oscillatory dynamics to evolve. A final interaction with the probe results in a four-wave-mixing signal which yields information about how the state has evolved during t_2.

The best way to represent 2DES data is to generate 2D maps which plot the signal as a function of excitation energy ($\hbar\omega_1$) and detection energy ($\hbar\omega_3$). Electronic transitions with inhomogeneous broadening show elongation of the signal along the diagonal ($\hbar\omega_1 = \hbar\omega_3$) at early times, whereas the antidiagonal line shape corresponds to the homogeneous width. The signals resulting from stimulated emission and photobleaching are positive in sign (red in our maps) and photoinduced absorption signals are negative (blue in our maps). Analysis of the t_2-dynamics is typically done by selecting a specific point ($\hbar\omega_1$, $\hbar\omega_3$) in the 2D map and plotting the corresponding signal amplitude as a function of t_2.

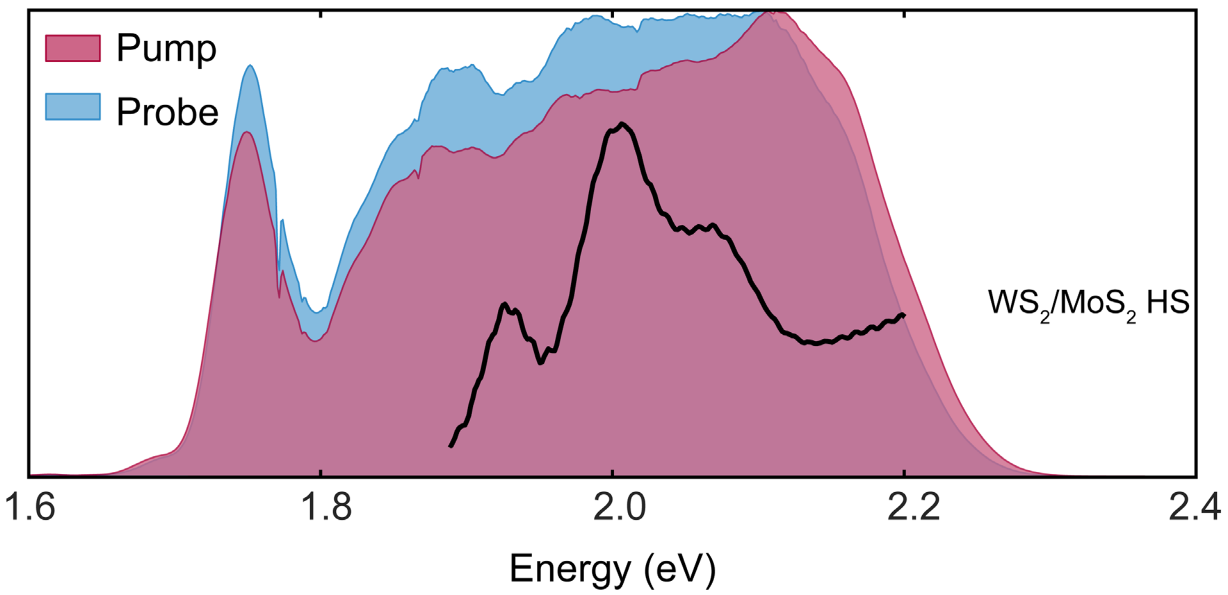

Figure S1. Absorption spectrum of the HS (black) plotted against experimental pump (purple) and probe (blue) spectra. Both pulses are generated from the same NOPA.

2DES setup

The home-built 2DES instrument generates a pair of time delayed pump pulses with pairs of birefringent wedges which scan the inter-pump pulse delay, t_1, with high phase stability[4]. The partially collinear beam geometry allows for direct collection of the real absorptive signal. Pump and probe beams are generated by the same non-collinear optical parametric amplifier (NOPA), seeded by a 1 kHz Ti:Sapphire regenerative amplifier (Libra, Coherent) generating 100 fs pulses at 1.55 eV photon energy. The NOPA is tuned with central photon energy $\hbar\omega = 2.0$ eV and 300 meV bandwidth and displays < 0.25% RMS fluctuations. The spectra of the pump and probe pulses are reported in Figure S1. The pulses are compressed down to sub-20-fs duration using a couple of chirped mirrors. Experiments were performed with pump and probe beams with orthogonal linear polarizations to suppress the pump scattering. Pump and probe beams are focused on the sample to diameters of 200 μm and 80 μm, respectively. The waiting time, t_2, is scanned in 5 fs steps from $-50<t_2<50$ fs followed by 10 fs steps up to 1 ps.

The pair of phase-locked pump pulses are generated using pairs of birefringent wedges in a device termed Translating-Wedge-Based Identical Pulses eNcoding System (TWINS)[4]. The pump pulse pair is generated with a 45° linearly polarized beam split into orthogonally polarized components after traveling through a series of birefringent α-barium borate plates and wedges. Two pairs of birefringent wedges are cut to selectively delay either the horizontally or the vertically polarized pump component. The t_1 delay between the pump pulses is controlled with a translation stage where one wedge of each pair is mounted. The delayed pump pair is projected back onto the 45° polarization with a linear polarizer[4]. In this study, t_1 is continuously scanned in the -30 fs $< t_1 <$ 220 fs range. A portion of the pump beam is sampled after traveling through a chopper; the sampled beam is sent to a photodiode which is used to calibrate the t_1 delay and to monitor the laser stability during the course of the experiment.

Pulse characterization

The instrumental response function (IRF) was measured using cross-correlated Polarization Gated Frequency-Resolved Optical Gating (PG-FROG). The pump and probe pulses are set with 45° relative polarization and focused into a 200-μm-thick glass plate at the sample position. Both beams pass through a 200-μm-thick fused silica plate mimicking the outer cryostat window to reproduce the dispersion of the pulses impinging on the sample. The probe is collected in a spectrometer with an analyzer set for 90° cross polarization with respect to the pump. The resulting PG-FROG trace for the WS_2/MoS_2 HS experiments is shown in Figure S2. The average full-width half maximum (FWHM) of the PG-FROG is 21.9 fs ± 3.4 fs (Figure S2a). Figure S2b shows the spectrogram of the IRF showing a negligible amount of linear and quadratic chirp across the main

region of interest (1.9-2.1 eV). The IRF offers an upper limit for the temporal resolution of the setup.

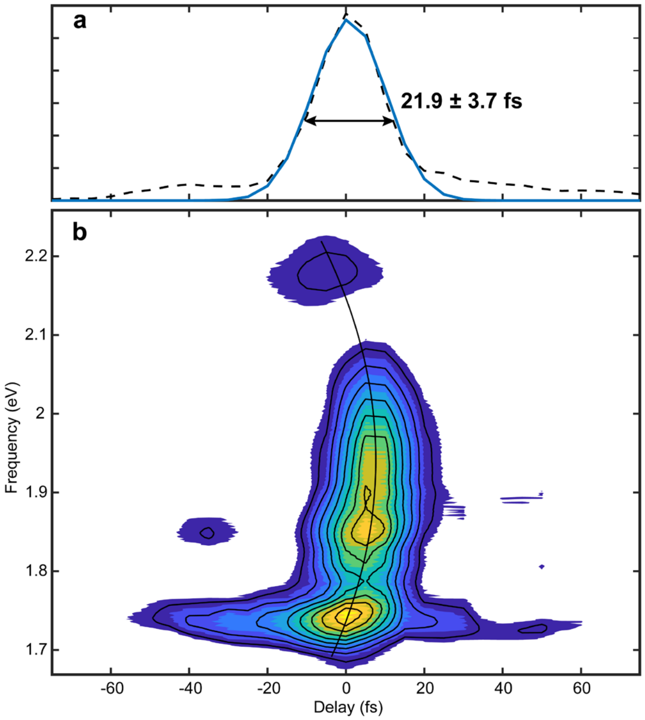

Figure S2. PG-FROG characterization of the pump and probe pulses. a) The marginal over the frequency axis fit with a Gaussian yields a 21.9 fs FWHM giving an upper limit for the experimental temporal resolution. b) The PG-FROG signal is shown as a function of pump-probe delay and detection frequency. The center of each peak amplitude as function of frequency estimates the overall chirp to be very weakly linearly (< 0.1 fs/meV) in addition to a weak quadratic contribution.

Large-Area Sample Preparation

MoS_2 and WS_2 monolayers are obtained by gold-assisted mechanical exfoliation from bulk MoS_2 (SPI Supplies) and WS_2 (HQ graphene)[7]. A gold layer is deposited on top of the bulk TMDs. When the gold layer is exfoliated away with a thermal release tape (Semiconductor corp.) it carries a large piece of TMD monolayer on the contact surface. Heating up at 130°C removes the thermal release tape and the residues are cleaned by acetone and O_2 plasma treatment. Finally, a gold etchant solution made from mixing KI (99.9%, Alfa Aesar) and I_2 (99.99%, Alfa Aesar) in

deionized water is used to dissolve gold. The TMD-monolayers are comparable to the monolayers obtained from conventional scotch tape exfoliation in terms of a clean surface and strong photoluminescence[7]. MoS_2 is first exfoliated and transferred onto a 200-μm-thick SiO_2 substrate. Then WS_2 is exfoliated and transferred on top of MoS_2/SiO_2.

The interlayer tilt angle of the WS_2/MoS_2 HS was determined via angularly resolved second harmonic generation (Figure S3). The SHG measurements were performed with a 800 nm pump from a 80 MHz repetition rate laser with approximately 3 mW of power resulting in a photon flux of about $1.51x10^8$ photons/pulse. Tight focusing (0.9 μm diameter) to the sample was achieved using a 100x objective lens with a numerical aperture of 0.75.

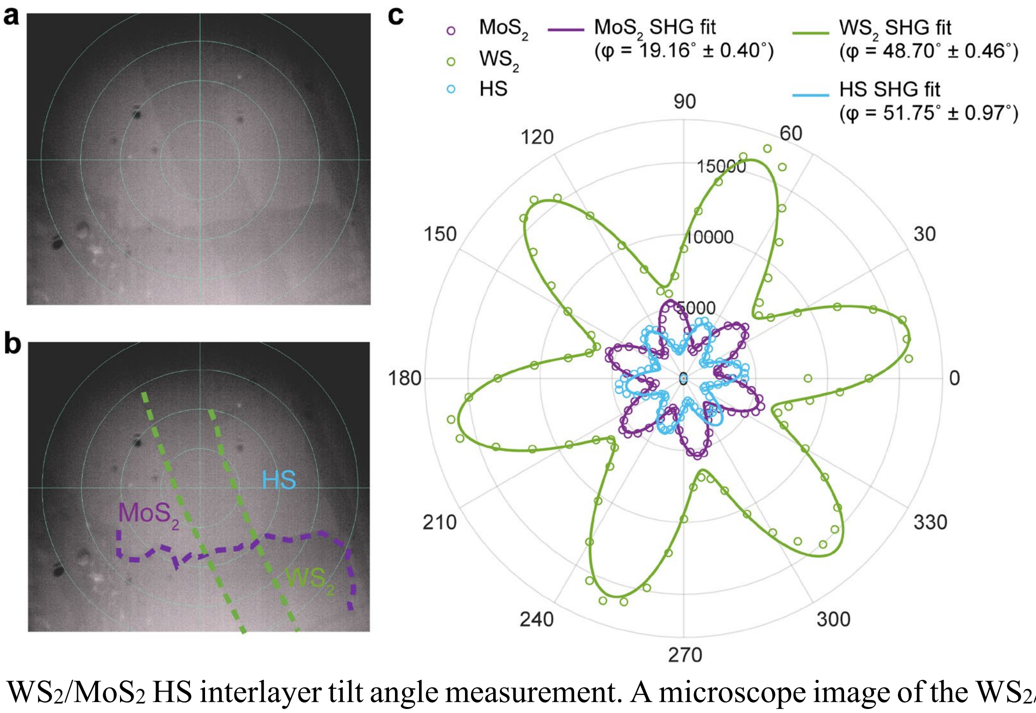

Figure S3 WS_2/MoS_2 HS interlayer tilt angle measurement. A microscope image of the WS_2/MoS_2 HS is shown for a 0.9 μm large region of the sample in (**a**) with the different layers highlighted in (**b**). **(c)** The interlayer tilt angle is determined from angularly resolved second harmonic generation (SHG) in three different spots on the sample. The difference between the SHG signal of the WS_2 and MoS_2 monolayers yields an interlayer tilt angle of 29.5°.

2DES map of isolated WS_2

2DES measurements were additionally performed on large-area WS_2 monolayer samples at 80 K (Figure S4) using the same pump and probe spectra as in the WS_2/MoS_2 HS and MoS_2 ML samples. For this reason, only the A_W exciton is resolved. At early t_2 the 2D maps is dominated by the strong diagonal peak of the A_W exciton which is significantly inhomogeneously broadened along the diagonal ($\hbar\omega_1 = \hbar\omega_3$) and shows somewhat narrow homogeneous broadening along the antidiagonal. At later times (t_2 = 200 fs) the elongated lineshape of A_W rounds out due to spectral diffusion and loss of correlation between excitation and detection events.

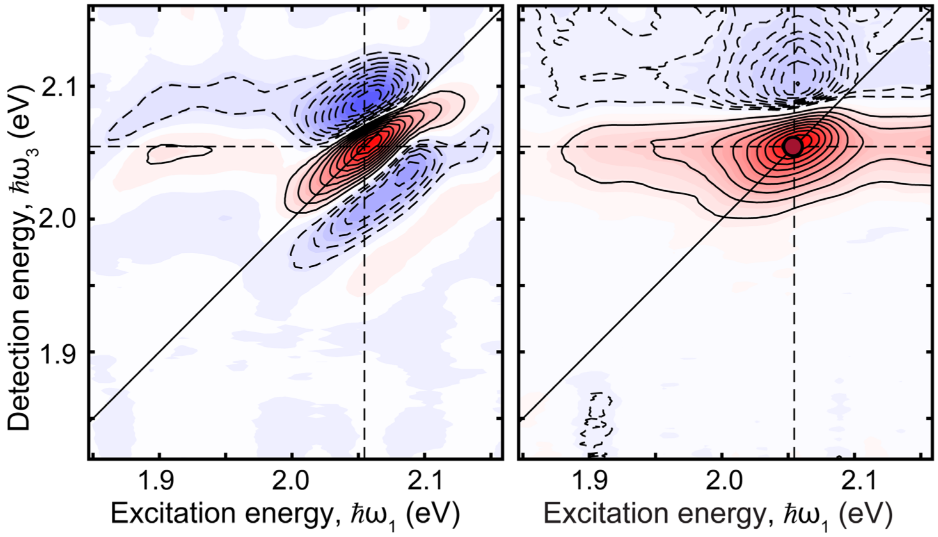

Figure S4. Real absorptive 2DES maps of WS_2 ML at t_2 = 0 fs (left) and t_2 = 200 fs (right). Dashed vertical and horizontal lines are drawn at the WS_2 A exciton energy at $\hbar\omega$ = 2 eV.

Fitting procedure and time constants

In order to characterize the kinetic behavior of interlayer charge transfer (ICT) we performed a simple fit to point-traces of the 2DES data. Point-traces were selected based on the exciton energies of each relevant sample for a specific frequency combination ($\hbar\omega_1$, $\hbar\omega_3$) and were then averaged over the surrounding 10 meV in the 2DES maps. The temporal dynamics of all the excitonic peaks of the 2DES maps measured on the HS and the isolated layers together with the corresponding fits are reported in Figure S5, S6 and S7.

The fitting function $F(t)$ is the product of a rising exponential and a single- or bi-exponential decay (depending on the dynamics):

$$F(t) = \left(1 - \exp\left(-\frac{t}{\tau_{rise}}\right)\right) * \left(A_1 \exp\left(-\frac{t}{\tau_1}\right) + A_1 \exp\left(-\frac{t}{\tau_2}\right)\right)$$

convoluted with a gaussian function representing the IRF. τ_{rise} and τ_1 (τ_2) are respectively the rise time and the decay time(s), while A_1 (A_2) are the weights of the two exponential functions. The full width at half maximum of the IRF is obtained by the PG FROG technique. The extracted fit

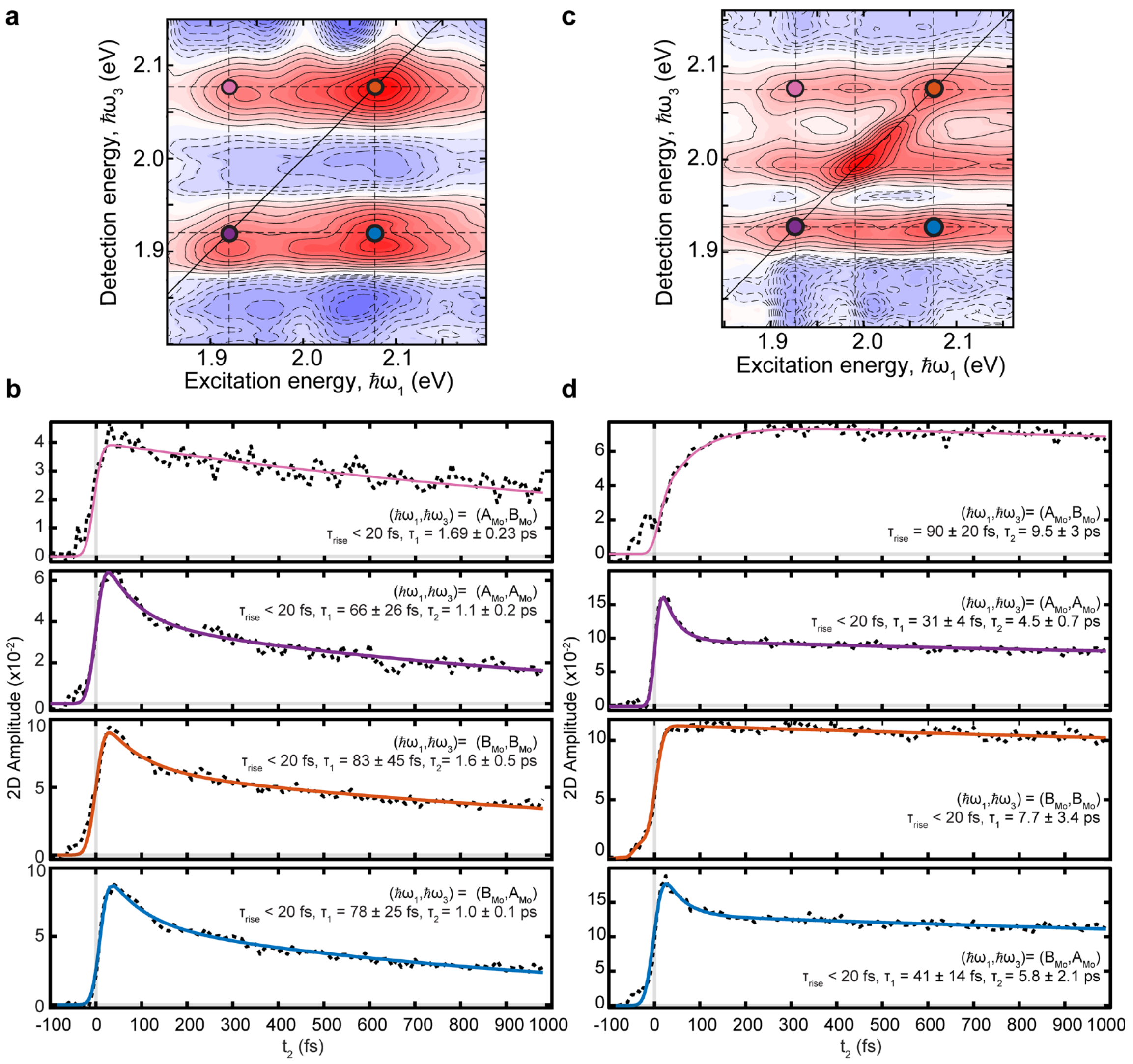

Figure S5: 2DES maps at t_2 = 200 fs and temporal dynamics of the diagonal and the cross-diagonal peaks measured at the energies of the A_{Mo} and B_{Mo} excitons for the isolated ML-MoS_2 **(a-b)** and the WS_2/MoS_2 HS **(c-d)**. t_2-dependent signals are averaged over 10 meV energy window. The continuous lines are the fit to the data.

coefficients are presented with the 95% confidence intervals. The build-up times τ_{rise} of the cross peaks dynamics in Figure 3b represent a precise estimation of the IHT and IET processes.

The 2DES measurements were performed on a limited temporal window (i.e. 1 ps) in order to achieve a high signal to noise ratio of the signals that allow us to perform high-quality fit of the temporal traces and to extract with accuracy the timescales of the early stage relaxation processes in the HS. For this reason, our fits can correctly reproduce only the formation and the fast (i.e. fs to ps) relaxation processes in TMDs while they cannot capture the long lived (i.e. hundreds of ps or ns) decay components expected for the radiative recombination process of interlayer excitons[8].

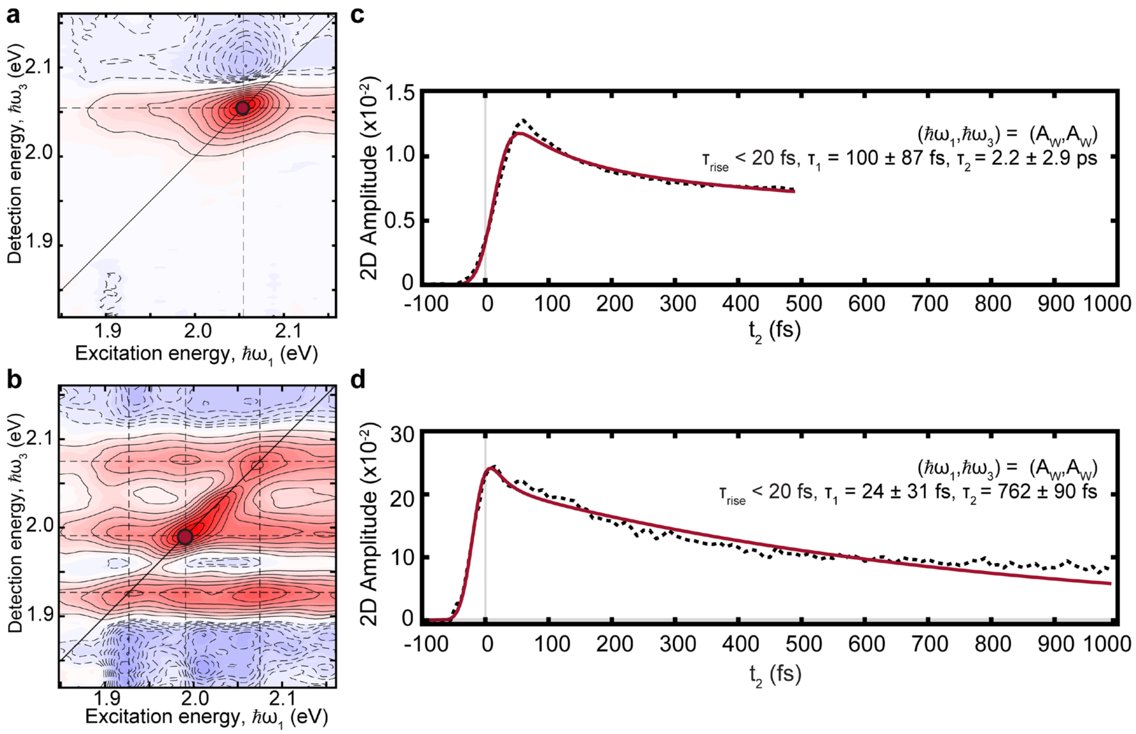

Figure S6: 2DES maps at t_2 = 200 fs and temporal dynamics of the diagonal A_W peak for the isolated ML-WS_2 **(a-b)** and the WS_2/MoS_2 HS **(c-d).** t_2-dependent signals are averaged over 10 meV energy window. The A_W exciton dynamics do not display any significant change.

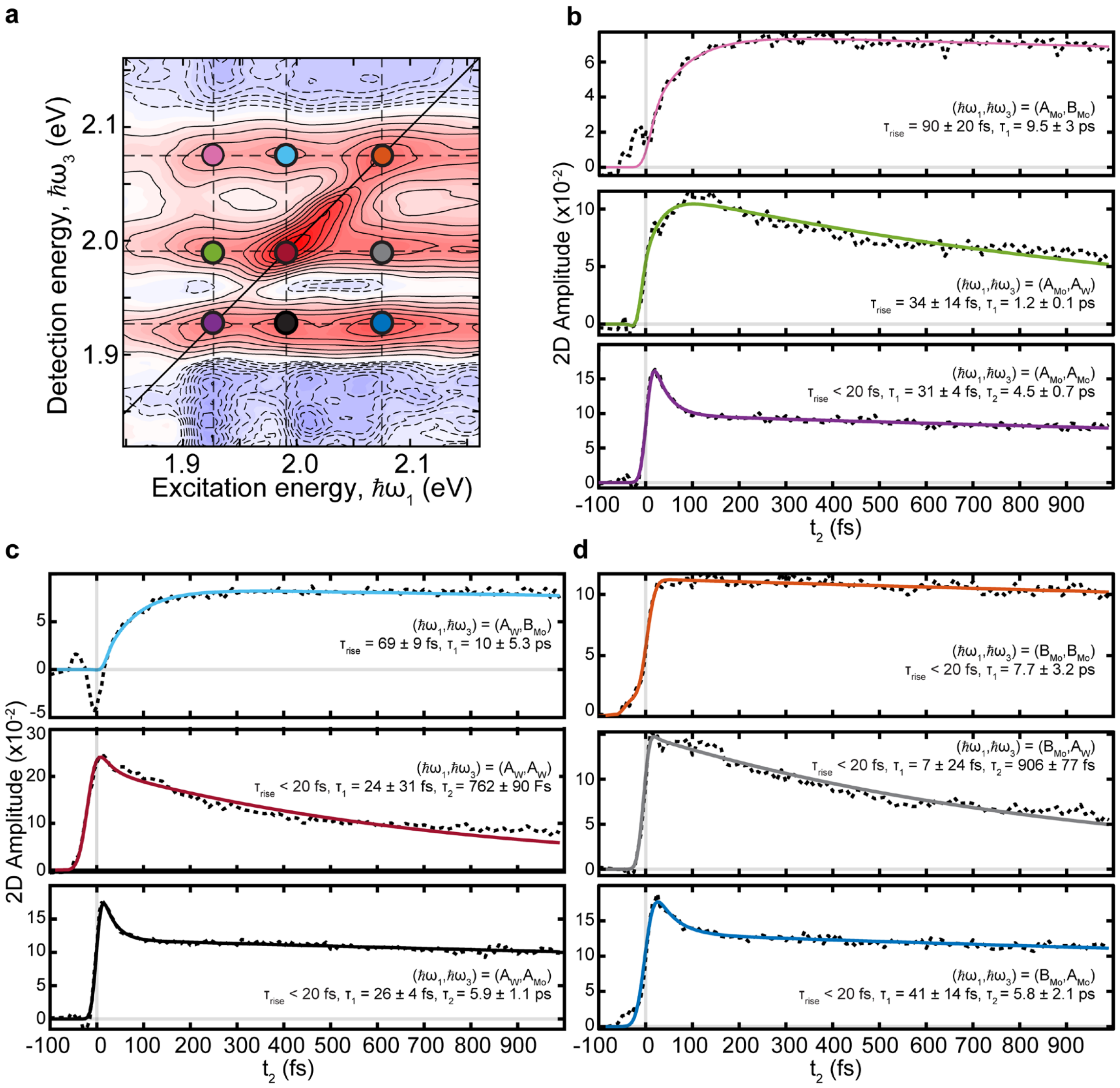

Figure S7. Fits to excitonic peaks of WS_2/MoS_2 HS. **(a)** Real absorptive 2DES map at $t_2 = 200$ fs for the WS_2/MoS_2 HS with color-coded dots depicting the ($\hbar\omega_1$,$\hbar\omega_3$) locations plotted as a function of t_2 in parts **(b-d)**. **(b-d)** t_2-dependent traces averaged over 10 meV (black, circles) with fit (color coded to match circles in a) arranged by the excitation frequency: $\hbar\omega_1 = 1.92$ eV **(b)**, 1.99 eV **(c)**, and 2.07 eV **(d)**. A finite (i.e. sub-100 fs) rise time is observed for above diagonal excitonic peaks ($\hbar\omega_1 < \hbar\omega_3$) due to the ICT processes. Temporal dynamics of below diagonal peaks ($\hbar\omega_1 > \hbar\omega_3$) are characterized by a pulsewidth limited build-up time. For these excitation/detection energies the PB exciton signals are dominated by the instantaneous Pauli blocking due to photoexcited carriers directly injected in MoS_2 and WS_2 layers.

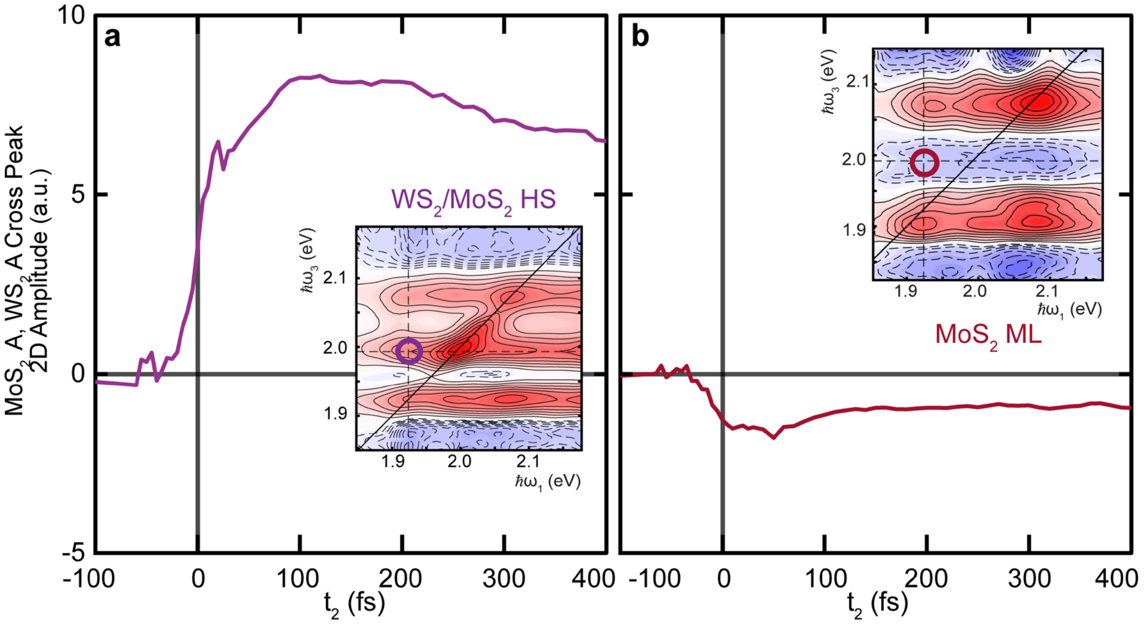

Figure S8. (A_{Mo}, A_W) cross-peak comparison. **(a)** The signature of delayed hole transfer (purple) is peculiar to the HS. **(b)** The exciton dynamics at the same spectral position in ML-MoS_2 (red) shows a completely different behavior: an instantaneous negative signal due to photoinduced renormalization of the exciton energy.

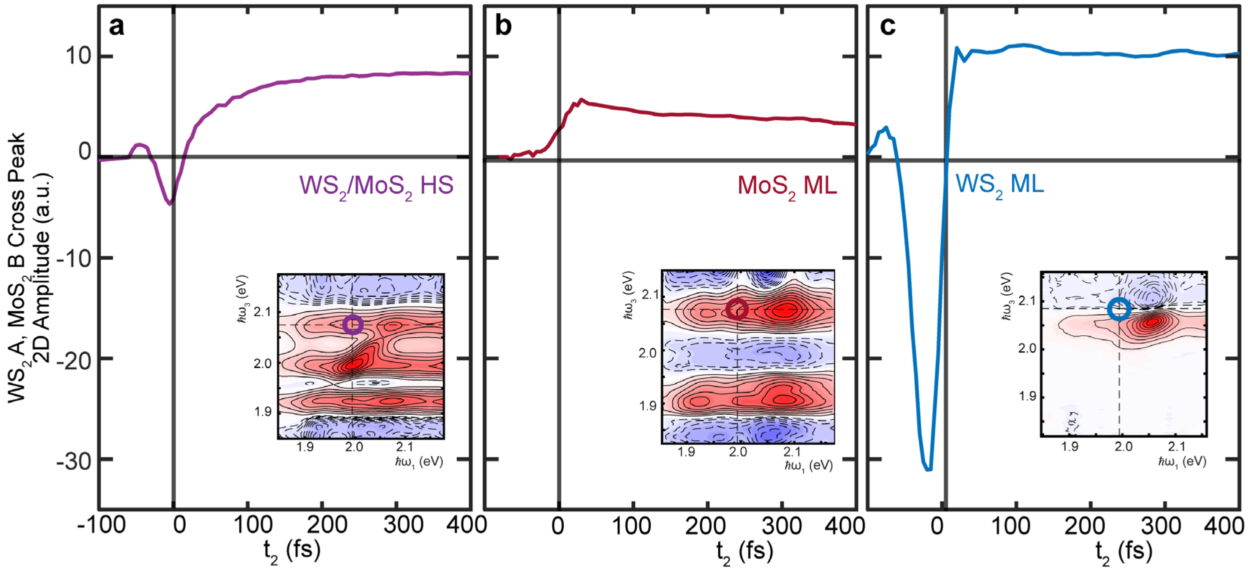

Figure S9. (A_W, B_{Mo}) cross-peak comparison. Temporal dynamics taken at the spectral location (ω_1,ω_3) = (A_W, B_{Mo}) measured on the WS_2/MoS_2 HS **(a)**, ML-MoS_2 **(b)**, and ML-WS_2 **(c)**. **(a)** The cross-peak dynamics in the HS is characterized by a slow rise attributed to the IET process. **(b)** In ML-MoS_2 the dynamics is characterized by an instantaneous rise due to the strong intralayer coupling between B_{Mo} and A_{Mo} excitons. **(c)** The same instantaneous dynamics is observed in ML-WS_2 and attributed to photoinduced renormalization of the optical gap. The strong negative feature at negative delays is attributed to a pump-perturbed free induction decay signal.

References

(1) Hybl, J. D.; Albrecht, A. W.; Gallagher Faeder, S. M.; Jonas, D. M. Two-Dimensional Electronic Spectroscopy. *Chem. Phys. Lett.* **1998**, *297* (3–4), 307–313.

(2) Jonas, D. M. Two-Dimensional Femtosecond Spectroscopy. *Annu. Rev. Phys. Chem.* **2003**, *54* (1), 425–463.

(3) Ogilvie, J. P.; Kubarych, K. J. Multidimensional Electronic and Vibrational Spectroscopy. In *Advances in Atomic, Molecular and Optical Physics*; 2009; Vol. 57, pp 249–321.

(4) Réhault, J.; Maiuri, M.; Oriana, A.; Cerullo, G. Two-Dimensional Electronic Spectroscopy with Birefringent Wedges. *Rev. Sci. Instrum.* **2014**, *85* (12), 123107.

(5) Fuller, F. D.; Ogilvie, J. P. Experimental Implementations of Two-Dimensional Fourier Transform Electronic Spectroscopy. *Annu. Rev. Phys. Chem.* **2015**, *66* (1), 667–690.

(6) Gallagher Faeder, S. M.; Jonas, D. M. Two-Dimensional Electronic Correlation and Relaxation Spectra: Theory and Model Calculations. *J. Phys. Chem. A* **1999**, *103* (49), 10489–10505.

(7) Liu, F.; Ziffer, M. E.; Hansen, K. R.; Wang, J.; Zhu, X. Direct Determination of Band-Gap Renormalization in the Photoexcited Monolayer MoS2. *Phys. Rev. Lett.* **2019**, *122* (24), 246803.

(8) Kiemle, J.; Sigger, F.; Lorke, M.; Miller, B.; Watanabe, K.; Taniguchi, T.; Holleitner, A.; Wurstbauer, U. Control of the Orbital Character of Indirect Excitons in MoS2/WS2 Heterobilayers. *Phys. Rev. B* **2020**, *101* (12), 121404.
